# Maternal satisfaction on quality of childhood vaccination services and its associated factors at public health centers in Addis Ababa, Ethiopia

**DOI:** 10.1186/s12913-023-10174-7

**Published:** 2023-11-29

**Authors:** Eyasu Tesfaye, Ayal Debie, Fasil Sisay, Tesfahun Zemene Tafere

**Affiliations:** 1 Department of Clinical Governance and Quality Management Office, Gandih Memorial Hospital, Addis Ababa, Ethiopia; 2 Department of Health Systems and Policy, Institute of Public Health, College of Medicine and Health Sciences, University of Gondar, P.O. Box: 196, Gondar, Ethiopia; 3 Department of Nursing, Menelik II Medical and Health Science College, Kotebe University of Education, Addis Ababa, Ethiopia

**Keywords:** Childhood vaccination, Ethiopia, Maternal satisfaction, Quality of service

## Abstract

**Background:**

Vaccination is one of the most important public health interventions to reduce child mortality and morbidity. In Ethiopia, about 472,000 children die each year by vaccine-preventable diseases. A satisfied mother is assumed to use the services and complies with the service provider for better health care outcomes. However, there was no adequate evidence regarding maternal satisfaction with quality of childhood vaccination services. This study aimed to assess maternal satisfaction on quality of childhood vaccination services and its associated factors at public health centers in Addis Ababa, Ethiopia.

**Methods:**

A facility-based cross-sectional study was conducted from 12 July to 12 August 2021 at public health centers in Addis Ababa, Ethiopia. A total of 366 mothers (caretakers) of under one-year-old children participated in the study. A systematic sampling technique with an interviewer-administered questionnaire and inventory checklist were used to collect the data. A binary logistic regression model was fitted. Adjusted Odds Ratio (AOR) with 95% confidence interval (CI) and *p*-value < 0.05 were used to identify the factors associated with the outcome.

**Results:**

Nearly two-thirds (61.2%) of mothers (caretakers) were satisfied with the quality of childhood vaccination services. Service providers’ greeting [AOR = 1.60; 95%CI: 1.37–1.99] and information about the types of vaccines [AOR = 1.54; 95%CI: 1.32–1.89] were positively associated with maternal satisfaction. On the contrary, long waiting time of mothers (caretakers) to receive services [AOR = 0.29; 95%CI: 0.14–0.62] was negatively associated with services.

**Conclusion:**

The overall maternal satisfaction towards the quality of childhood vaccination services in this study was found to be low. Minimizing waiting time at the health facility, enhancing greetings and providing adequate information regarding childhood vaccination for mothers (caretakers) improved their satisfaction with the services.

## Background

In 2015, about six million children died globally before the age of five, and over 3 million are died from vaccine-preventable infectious diseases [1]. In Ethiopia, about 472,000 children still die each year from vaccine-preventable diseases [2]. Majorities of childhood deaths are due to diseases that could be easily prevented with simple and affordable interventions such as vaccines [3]. Children are more susceptible to certain pathogens including vaccine-preventable infectious diseases due to their weak immunity [4]. As a result, vaccination is one of the most powerful health interventions that prevent 2–3 million deaths every year from vaccine-preventable diseases [2].

Vaccination is the process by which an individual’s immune system becomes fortified against an infectious disease by introducing a vaccine into the body that triggers an immune response [5]. Vaccines contain only killed or weakened forms of microorganisms like viruses or bacteria that do not cause the disease [6]. Vaccination is a key component of primary health care and a proven tool in preventing and eradicating infectious diseases [7]. It is a simple, safe and effective way of preventing and controlling infectious disease outbreaks [8, 9]. As a result, childhood immunization is considered a priority child health service in health facilities[10]. However, about 60% of children did not receive quality childhood vaccination services, particularly in sub-Saharan African countries [11].

The quality of childhood vaccination service depends on three domains: Structure, process, and outcome [12]. The driving forces towards the quality of childhood vaccination services include a clear understanding of the benefits of vaccination among community members, a readiness for providing vaccination by the health workers, and interventions to overcome barriers to vaccination services [13]. The quality of childhood vaccination services is also linked to the unavailability of vaccines, poor cold chain management, long waiting times, fear of side effects, long-distance travel, and being busy [14]. Maternal (caretaker) satisfaction is considered one of the most frequently used proxy indicators to measure the quality and efficiency of childhood vaccination services [11]. Accordingly, poor quality of childhood vaccination services is a major cause of morbidity and mortality of children due to vaccine-preventable diseases in developing countries[15] This can also cause a reduction in productivity, educational achievements, and economic growth [16]. A low level of maternal satisfaction with the quality of vaccination services may also enhance the self-medication of children using antibiotics associated with childhood illness among non-vaccinated children [12]. Even though there are studies in Ethiopia that evaluated the coverage of childhood immunization services [17, 18], there were no adequate evidences regarding maternal satisfaction on the quality of childhood vaccination services. Therefore, this study aimed to assess maternal satisfaction with the quality of childhood vaccination services and its associated factors at public health centers in Addis Ababa, Ethiopia.

## Methods and materials

### Study design, setting, and population

A facility-based cross-sectional study was conducted from 12 July to 12 August 2021 at public health centers in Addis Ababa, Ethiopia. Addis Ababa is the capital city of Ethiopia and is the largest city in the country. According to the city administrative health department biannual profile report, the city has 11 sub-cities, 12 public hospitals and 109 public health centers, and a total population of 4,567,857. The total number of eligible populations in the city who attended the expanded program of immunization (EPI) clinic in public health centers was about 13,500 per month.

### Population

All mothers (caretakers) of under one year of children who attended childhood vaccination services at health centers in Addis Ababa city administration were the source population while those mothers (caretakers) who attended vaccination services for their children in the selected health centers were the study population. All mothers (caretakers) of under one year of children who were receiving immunization services at least one schedule within the selected sub-cities of Addis Ababa were included in the study. Mothers (caretakers) of under one year of children who were, severely ill and unable to respond were excluded from the study.

### Sample size and sampling procedures

The sample size was determined using a single population proportion formula. It was computed by considering that a previous study has demonstrated the proportion of maternal satisfaction towards the quality of childhood vaccination service (68.2%) [19], a 95% confidence interval, 5% margin of error, and 10% non-response which yields the final sample size 366.

Four sub-cities and 33 health centers from these sub-cities were selected using simple random sampling. Then, the proportional allocation was done to each selected health center based on the previous one-month vaccination performances. Finally, a systematic sampling technique was employed and study participants were selected in every 3^rd^ interval.

### Variables and measurements

The dependent variable was maternal satisfaction on the quality of childhood vaccination service while sociodemographic status, infrastructures, process, and access factors were the independent variables. Maternal satisfaction with the quality of childhood vaccination service was measured in eight items, each containing a five-point Likert scale (1 = very dissatisfied to 5 = very satisfied) alternatives. Therefore the total score could be ranged from 8–40 and then we used the demarcation threshold formula: $((\frac{\mathrm{Total}\;\mathrm{highest}\;\mathrm{score}\;-\;\mathrm{Total}\;\mathrm{lowest}\;\mathrm{score}}2)\;+\;\mathrm{Total}\;\mathrm{lowest}\;\mathrm{score})$ [20] to categorize the level of mothers(caretakers) satisfaction as “satisfied and dissatisfied”. Accordingly, mothers (caretakers) who scored above 60% on the satisfaction measurement tools were considered as satisfied otherwise not dissatisfied.

### Data collection tools and procedures

A pre-tested interviewer-administered structured questionnaire was used to collect data. The data collection tool was adapted by reviewing a variety of literature [1, 21]. Furthermore, the Ethiopian Ministry of Health (MoH) inventory checklist was used to collect data related to the structure factors and process factors. The data collection tool was first prepared in English, translated to Amharic (the local language) then retranslated to English to check for consistency. Data collection was facilitated by four trained BSC nurses and two BSC midwifery nurse supervisors.

### Data quality assurance and control

A day of training was given for both data collectors and supervisors on the data collection methods and procedures. The data collectors were followed by supervisors daily. To enhance instrument reliability, the instrument was pre-tested on 5% of sampled mothers (caretakers) of under one year children outside of the study area nearby healthcare facilities with similar characteristics to those in the study. The data was collected after a brief explanation of the aim of the study to the participants. The validity and completeness of the collected data were verified by the principal investigator and supervisors daily.

### Data processing and analysis

Data were cleaned and entered into Epi info version 7.2.2.2 and exported to SPSS version 23 statistical software for analysis. A binary logistic regression was fitted to determine the association between dependent and independent variables. Variables with a *p*-value of less than 0.2 during bivariable regression were entered into multivariable logistic regression. Adjusted Odd Ratio (AOR) with a 95% confidence interval and *p*-value < 0.05 were used to declare the significant factors and strength of association. The model fitness was checked using the Hosmer Lemeshow goodness of fit test (88.6%) indicating that the model fitness was good. Descriptive statistics such as frequency, percentages, mean, and standard deviation (SD) were presented in the form of texts and tables.

## Results

### Socio-demographic characteristics

A total of 366 mothers (caretakers) of under one year children participated with a response rate of 100%. The mean age of mothers/caretakers was (25.6 ± 6.7 SD) years. More than one-third (41.5%) of the participants were housewives and 85.4% were married (Table 1).

**Table 1:** Socio-demographic characteristics of mothers (caretakers) attending EPI service at public health centers in Addis Ababa, Ethiopia, 2021 (*n* = 366)

Characteristics	Category	Frequency (n)	Percent (%)
Age (in year)	15–19	14	3.8
	20–24	93	25.4
	25–29	199	54.4
	30–34	58	15.8
	> 35	2	0.5
Ethnicity	Oromo	126	34.4
	Amhara	109	29.8
	Gurgae	49	13.4
	Others	82	22.4
Religion	Orthodox	208	56.8
	Muslim	101	27.6
	Others	57	15.6
Educational status	Not educated	24	6.6
	Primary (1–8)	98	26.8
	Secondary (9–12)	108	29.5
	College diploma and above	136	37.2
Currently marital status	Married	313	85.4
	Unmarried	53	14.6
Occupational status	Housewife	152	41.5
	Government employee	97	26.5
	Merchant	117	32.0
Monthly income (US$)	< 24.11	85	23.2
	24.11- 48.23	65	17.8
	48.23–96.46	95	26.0
	> 96.46	121	33.1

### Availability of infrastructures

All health centers had separate immunization rooms and 97.0% of them had a separate waiting area with chairs. Most health centers (97.0%) had adequate immunization registration books and tally sheets. Less than three-fourths (72.7%) of the health centers had pipe water. All health centers had at least one refrigerator whereas, 21.2% of health centers did not have a separate room for refrigerators and cold boxes (Table 2).

**Table 2:** Availability of organizational infrastructures for mothers receiving immunization service at public health centers in Addis Ababa, Ethiopia, 2021 (*n* = 33)

Inventory checklist	Status	
	**Yes (%)**	**No (%)**
Separate waiting area	32 (97.0)	1 (3.0)
Separate vaccination room	33 (100)	0
Waste disposal system (safety box, plastic bag, bucket, and inclinators)	30 (90.9)	3 (9.1)
Talley sheets for the last six months	32 (97.0)	1 (3.0)
Immunization card for the last six months	32 (97.0)	1 (3.0)
Standard registration book	32 (97.0)	1 (3.0)
Availability of all types of syringes	32 (97.0)	1 (3.0)
Availability of all types of vaccine	31 (93.9)	2 (6.1)
Availability of diluents and droppers	31 (93.9)	2 (6.1)
Availability of pipe water in the units	24 (72.7)	9 (27.3)
Availability of last quarter integrative supportive supervision feedback given	11 (33.3)	22 (66.7)
Cleanness of room before start service	27 (81.8)	6 (18.2)
Providers' greeting to health seekers	3 (9.1)	30 (90.9)
The vaccination monitoring chart was filled and plotted	32 (97.0)	1 (3.0)
Providers wash his/her hands before and after the administration of vaccines	1 (3.0)	32 (97.0)
Record and documentation	30 (90.9)	3 (9.1)
Providers correctly assess which infants are eligible for vaccines	31 (93.9)	2 (6.1)
Healthcare providers explain the dose and type of vaccines	4 (12.1)	29 (87.9)
Providers use an ice pack, and vaccine carriers correctly	32 (97.0)	1 (3.0)
Providers correctly reconstitute the antigen with appropriate dilute as needed	33 (100)	0
Providers use safety boxes to dispose of needles appropriately	21 (63.6)	12 (36.4)
Providers dispose of vials, plastics, and swabs correctly	26 (78.8)	7 (21.2)
Providers did not recap the needle after administering vaccines	32 (97.0)	1 (3.0)
Functional refrigerator and cold box	33 (100)	0
Separate room for refrigerator and cold box	21 (63.6)	12 (21.2)
Providers use vaccine carriers and cold boxes correctly	33 (100)	0
Vaccine kept under appropriate compartment	33 (100)	0
The temperature of the refrigerator was recorded twice per day	30 (90.9)	3 (9.1)

#### Access to vaccination services

The majority (80.9%) of mothers (caretakers) judged that the service was located at a convenient distance from their home. Moreover, 39.1% of mothers waited more than 45 min to receive the services (Table 3).

**Table 3:** Access to vaccination services for mothers receiving immunization services at public health centers in Addis Ababa, Ethiopia, 2021 (*n* = 366)

Accessibility factors	Categories	Respondents Status	
		**Frequency (n)**	**Present (%)**
Duration of time to reach the health facility on foot (minutes)	< 30 min	318	86.9
	≥30 min	48	13.1
Distance to home	< 5 km	296	80.9
	≥5 km	70	19.1
Mode of transportation	Foot	95	26.0
	Bajaj/Taxi	271	74.0
Waiting time to receive the service	< 15 min	56	15.3
	15-30 min	53	14.5
	30-45 min	114	31.1
	> 45 min	143	39.1
Working hour is convenient	Yes	304	83.1
	No	62	16.9

#### Process-related factors to vaccination services

Three-fourths (76.0%) of mothers (caretakers) had gotten vaccination services as per their previous appointments. About two-thirds (69.9%) of mothers (caretakers) were informed about the use and side effects of the administrated antigens but only 35% of them were informed about the types of vaccines given to their child. About 36.9% of mothers (caretakers) had gotten a proper greeting from the health care providers during their child vaccination session (Table 4).

**Table 4:** Process-related factors for mothers receiving immunization services at public health centers in Addis Ababa, Ethiopia, 2021 (*n* = 366)

**Process factors**	**Yes (%)**	**No (%)**
Cleanliness of the waiting area	288 (78.7)	78 (21.3)
The child received vaccination services as per their appointment	278 (76.0)	88 (24.0)
Healthcare providers greet service users	135 (36.9)	231 (63.1)
Information about the types of the current vaccine/s given to the child	128 (35.0)	238 (65)
Providers told clients about their next appointment	345 (94.3)	21 (5.7)
Information is given about the use and side effects of the administered antigen	256 (69.9)	110 (30.1)
Healthcare workers tell the doses of the vaccine	15 (4.1)	351 (95.9)

### Maternal satisfaction and its associated factors

Nearly two-thirds (61.2%) of mothers (caretakers) were satisfied with the overall quality of childhood vaccination services. Two-thirds (65.3%) and 61.2% of mothers/caretakers were satisfied with the cleanliness and availability of services, respectively. On the other hand, over half (52.0%) of mothers/caretakers were dissatisfied with access to drinking water, latrine, and hand-washing facilities (Table 5).

**Table 5:** Maternal satisfaction on the quality of childhood vaccination services at public health centers in Addis Ababa, Ethiopia, 2021 (*n* = 366)

Items	VS n (%)	S n (%)	N n (%)	DS n (%)	VDS n (%)
Satisfaction with the availability of service based on the previous appointment	89 (24.3)	224(61.2)	15 (4.1)	33 (9.0)	5 (4.1)
Satisfaction with time spent in the waiting room	49 (13.4)	176(48.1)	15 (4.1)	115 (31.4)	11 (3.0)
Satisfaction with the availability of providers at working time	48 (29.5)	223(60.9)	7 (1.9)	82(22.4)	6 (1.6)
Satisfaction with the cleanliness of the vaccination room	39 (10.7)	239(65.3)	239 (6.3)	59 (16.1)	6 (1.6)
Satisfaction with the friendliness (politeness) of the provider	28(7.7)	170(46.4)	51 (13.9)	105(28.7)	12 (3.3)
Satisfaction with the information given regarding vaccines and benefits of vaccinations	45 (12.3)	226(61.7)	24 (6.6)	60 (16.4)	11 (3.0)
Access to drinking water, latrine, and hand-washing facility	29 (7.9)	147(40.2)	50 (13.7)	125(34.2)	15 (4.1)
Satisfaction with the knowledge of the provider	25(6.8)	195(53.3)	109(29.8)	31(8.5)	6 (1.6)

*VS* Very Satisfied, *S* Satisfied, *N* Neutral, *DS* Dissatisfied, *VDS* Very Dissatisfied

### Factors associated with maternal satisfaction

In the final multivariable logistic regression analysis, waiting time at the vaccination unit service provider greetings, and information about the types of vaccines were factors associated with mothers' (caretakers) satisfaction with the quality of vaccination services. The odds of client satisfaction with the quality of services among mothers (caretakers) waiting 30–45 min to receive services is significantly lower as compared to those who waited below 15 min [AOR = 0.38; 95%CI: 0.19–0.78]. Moreover, the odds of client satisfaction with the quality of services among mothers (caretakers) who had got greeting from providers was 1.60 times higher [AOR = 1.60; 95%CI: 1.37–1.99] as compared with their counterparts. Mothers (caretakers) who had got information about the types of vaccines were satisfied 1.54 times [AOR = 1.54; 95%CI: 1.32–1.89] compared with those who did not get information about the types of vaccines (Table 6).

**Table 6:** Factors associated with maternal satisfaction towards the quality of childhood vaccination services at public health centers in Addis Ababa, Ethiopia, 2021(n = 366)

**Characteristics**	**Maternal satisfaction**		**COR (95%CI)**	**AOR (95%CI)**
	**Satisfied**	**Dissatisfied**		
**Occupations**				
Housewife	89	60	1	1
Government employee	78	39	1.34 (0.81–2.23)	1.24 (0.73–2.11)
Merchant	57	43	1.50 (0.86–2.62)	1.32 (0.73–2.38)
**Distance to home**				
< 5 km	186	110	0.70 (0.41–1.18)	0.57 (0.32–1.03)
≥ 5 km	38	32	1	1
**Waiting time at the vaccination unit**				
< 15 min	36	17	1	1
15-30 min	43	13	0.33 (0.16–0.67)	0.29 (0.14–0.62)^a^
30-45 min	70	44	0.52 (0.26–1.01)	0.38 (0.19–0.78)^a^
> 45 min	75	68	0.69 (0.42–1.14)	0.63 (0.37–1.08)
**Working hours convenience**				
Yes	44	18	1.68 (0.93–3.05)	1.52 (0.81–2.84)
No	180	124	1	1
**Service provider greetings**				
Yes	91	44	1.65 (1.21–2.02)	1.60 (1.37–1.99)^a^
No	133	98	1	1
**Vaccination service as per their appointment**				
Yes	174	104	0.78 (0.48–1.28)	1.03 (0.61–1.75)
No	50	38	1	1
**Information about types of vaccines**				
Yes	88	40	1.60 (1.38–1.95)	1.54 (1.32–1.89)^a^
No	136	102	1	1
**Information on uses and side effects of the vaccine**				
Yes	162	94	0.74 (0.47–1.18)	0.85 (0.52–1.38)
No	62	48	1	1
**Cleanliness of the waiting area**				
Yes	183	105	0.63 (0.38–1.05)	0.78 (0.42–1.27)
No	41	37	1	1

^a^Significant, *AOR* Adjusted Odd Ratio, *COR* Crude Odd Ratio, *CI* Confidence Interval

## Discussion

The overall maternal satisfaction towards the quality of childhood vaccination services in this study was found to be low. We also found that the major constraints that determine the satisfaction of mothers (caretakers) towards vaccination services were waiting time at the vaccination unit, service provider greetings, and information about the types of vaccines were factors associated with mothers' (caretakers’) satisfaction towards the quality of vaccination services.

Moreover, the finding of this study was relatively similar to the finding from Ondo State in Nigeria and North Wollo, Ethiopia which reported that the overall maternal satisfaction towards the quality of childhood vaccination services was (68.2%) and (68.9%) respectively [22, 19]. This consistency might be due to the similarity of the vaccination services delivery system.

Furthermore, the finding of this study was relatively higher as compared with the finding reported from Egypt (63%) [23], Calabar, south Nigeria (43.6%) [24], and Jigjiga, Ethiopia (30.1%) [25], However, the finding of this study was lower when compared with findings from New Cairo district, Egypt (74.4%) [26], Suez governorate, Egypt (95.2%) [27], Sokoto metropolis, Nigeria (75%) [28], Kombolicha, Ethiopia (71.9%) [21] and Hawasa city, Ethiopia (76.7%) [20]. This variation might be due to the existence of differences in cultural context towards vaccination services, and service delivery systems. Moreover, differences in the provision of refreshment training on vaccination services and healthcare providers' tendency towards using vaccination services guidelines and protocols might be attributed to the observed differences.

In this study, a long waiting time to find the services was found to be negatively influenced mothers' (caretakers) satisfaction. Mothers (caretakers) who had a waiting time of 30–45 min for service at the vaccination unit were less likely to be satisfied with the quality of childhood vaccination services compared with their counterparts. This finding was similar to the finding in Wadla district, Ethiopia [19], Kombolicha, Ethiopia [21], Jigjiga, Ethiopia [25], and India [14]. The possible justification might be because serving with short waiting time might be linked with accessing the services early and this could also increase the satisfaction of mothers (caretakers) towards the quality of childhood vaccination services.

Moreover, a friendly relationship between health care providers and mothers (caretakers) positively influenced mothers’ (caretakers’) satisfaction. Mothers (caretakers) who got greetings from providers were more likely to be satisfied with the quality of childhood vaccination services compared with mothers (caretakers) who did not get greetings. This finding was supported by the findings in Wadla district, Ethiopia [19], and Kombolicha, Ethiopia [21]. The possible justification might be due to effective interaction between service providers and service caretakers can contribute to the motivation of mothers' acceptance towards the vaccine services and it also increases the satisfaction of mothers with childhood vaccination services.

Information about the types of vaccines was also a significant predictor of maternal satisfaction with the quality of childhood vaccination services. Mothers (caretakers) who got information about the types of vaccines were 1.54 times more likely to be satisfied with the quality of childhood vaccination services than those who did not have information. The finding of this study was similar to a study conducted in Wadla district, Ethiopia [19], Jigjiga, Ethiopia [25] and Kombolcha city, Ethiopia [21]. This might be because having proper information about the types of vaccines enhances mothers' awareness so that they may have acceptance towards the services.

## Limitations of the study

The limitation of this study is that it lacked qualitative aspects in assessing maternal satisfaction with the quality of childhood vaccination services and its associated factors. The other possible limitation of the study was that there might be a social desirability bias.

## Conclusion

The maternal satisfaction with the quality of childhood vaccination services in this study was low. Long waiting time to find the services was found to be negatively influenced mothers' (caretakers) satisfaction. Moreover, a friendly relationship between health care providers and mothers (caretakers) positively influenced mothers' (caretakers') satisfaction. Precise and adequate information about the types of vaccines was also a positive predictor of maternal satisfaction with the quality of services.

Therefore, health sectors at each level and non-governmental organizations should work to improve the accessibility of services to caretakers. Healthcare providers should provide adequate information to mothers (caretakers) about vaccines and should have friendly interactions with mothers (caretakers). Moreover, healthcare providers had better adhere to vaccination services protocol. We also recommended future researchers to evaluate the quality of the services using a qualitative approach to get more general information.

## Data Availability

Data will be available upon reasonable request from the corresponding author.

## Abbreviations

AOR: Adjusted Odd Ratio
CI: Confidence Interval
EPI: Expanded Program on Immunization
SPSS: Statistical Package for Social Sciences
SD: Standard Deviation
WHO: World Health Organization

## References

[1] GebreEyesus FA, Assimamaw NT, GebereEgziabher NT, Shiferaw BZ (2020). Maternal satisfaction towards childhood immunization service and its associated factors in Wadla District, North Wollo, Ethiopia, 2019. Int J Pediatr.

[2] Meleko A, Geremew M, Birhanu F (2017). Assessment of child immunization coverage and associated factors with full vaccination among children aged 12–23 months at Mizan Aman town, bench Maji zone, Southwest Ethiopia. Int J Pediatr.

[3] Abadura SA, Lerebo WT, Kulkarni U, Mekonnen ZA (2015). Individual and community level determinants of childhood full immunization in Ethiopia: a multilevel analysis. BMC Public Health.

[4] Desalew A, Semahegn A, Birhanu S, Tesfaye G (2020). Incomplete vaccination and its predictors among children in Ethiopia: a systematic review and meta-analysis. Global Pediatr Health.

[5] Eshete A, Shewasinad S, Hailemeskel S (2020). Immunization coverage and its determinant factors among children aged 12–23 months in Ethiopia: a systematic review, and meta-analysis of cross-sectional studies. BMC Pediatr.

[6] Wondimu A, Cao Q, Asuman D, Almansa J, Postma MJ, van Hulst M (2020). Understanding the improvement in full childhood vaccination coverage in Ethiopia using Oaxaca–blinder decomposition analysis. Vaccines.

[7] Chanie MG, Ewunetie GE, Molla A, Muche A (2021). Determinants of vaccination dropout among children 12–23 months age in north Gondar zone, northwest Ethiopia, 2019. PLoS ONE.

[8] Lakew Y, Bekele A, Biadgilign S (2015). Factors influencing full immunization coverage among 12–23 months of age children in Ethiopia: evidence from the national demographic and health survey in 2011. BMC Public Health.

[9] Theodros G, Ibrahim K, Abebe B, Atkure D, Mekonnen T, Habtamu T, Kassahun A, Terefe G, Yibeltal A, Amha K (2017). Child health service provision in Ethiopia: outpatient, growth monitoring and immunization. Ethiop J Health Dev.

[10] Adamu AA, Uthman OA, Wambiya EO, Gadanya MA, Wiysonge CS (2019). Application of quality improvement approaches in health-care settings to reduce missed opportunities for childhood vaccination: a scoping review. Hum Vaccin Immunother.

[11] Ropero-Álvarez AM, El Omeiri N, Kurtis HJ, Danovaro-Holliday MC, Ruiz-Matus C (2016). Influenza vaccination in the Americas: progress and challenges after the 2009 A (H1N1) influenza pandemic. Hum Vaccin Immunother.

[12] Klugman KP, Black S (2018). Impact of existing vaccines in reducing antibiotic resistance: primary and secondary effects. Proc Natl Acad Sci.

[13] Abodunrin O, Adeomi A, Adeoye O (2014). Clients’ satisfaction with quality of healthcare received: Study among mothers attending infant welfare clinics in a semi-urban community in South-western Nigeria. Sky J Med Med Sci.

[14] Tesema GA, Tessema ZT, Tamirat KS, Teshale AB (2020). Complete basic childhood vaccination and associated factors among children aged 12–23 months in East Africa: a multilevel analysis of recent demographic and health surveys. BMC Public Health.

[15] Streefland PH (2001). Public doubts about vaccination safety and resistance against vaccination. Health Policy.

[16] Abdulrahman M, Habeeb Q, Teeli R (2020). Evaluation of child health booklet usage in primary healthcare centres in Duhok Province. Iraq Public Health.

[17] Nour TY, Farah AM, Ali OM, Abate KH (2020). Immunization coverage in Ethiopia among 12–23 month old children: systematic review and meta-analysis. BMC Public Health.

[18] Mohamud AN, Feleke A, Worku W, Kifle M, Sharma HR (2014). Immunization coverage of 12–23 months old children and associated factors in Jigjiga District, Somali National Regional State. Ethiopia BMC Public Health.

[19] GebreEyesus FA, Assimamaw NT, GebereEgziabher NT, Shiferaw BZ (2020). Maternal satisfaction towards childhood immunization service and its associated factors in Wadla District, North Wollo, Ethiopia, 2019. Int J Pediatr.

[20] Dana E, Asefa Y, Hirigo AT, Yitbarek K (2021). Satisfaction and its associated factors of infants’ vaccination service among infant coupled mothers/caregivers at Hawassa city public health centers. Hum Vaccin Immunother.

[21] Hussien A (2015). Assessment of Maternal Satisfaction Towards Childhood Immunization and Its Associated Factors in MCH Clinic, at Kombolcha, in Amhara Regional State.

[22] Babalola TK. Client satisfaction with maternal and child health care services at a public specialist hospital in a Nigerian Province. Turk J Public Health. 2016;14(3).

[23] El Gammal HAAA (2014). Maternal satisfaction about childhood immunization in primary health care center, Egypt. Pan Afr Med J.

[24] Udonwa N, Gyuse A, Etokidem A, Ogaji D (2010). Client views, perception and satisfaction with immunisation services at Primary Health Care Facilities in Calabar, South-South Nigeria. Asian Pac J Trop Med.

[25] Salah AA, Baraki N, Egata G, Godana W (2015). Evaluation of the quality of Expanded Program on immunization service delivery in primary health care institutions of Jigjiga Zone Somali Region, Eastern Ethiopia. Euro J Prev Med.

[26] Ebeid Y (2016). The acceptability of the Family Health Model, that replaces Primary Health Care, as currently implemented in Wardan Village, Giza, Egypt.

[27] El HAAAR. Maternal satisfaction about childhood immunization in primary health care center, Egypt. Pan Afr Med J. 2014;18. 10.11604/pamj.2014.18.157.1773 PMC4236845 25419295

[28] Timane AJ, Oche OM, Umar KA, Constance SE, Raji IA (2017). Clients' satisfaction with maternal and child health services in primary health care centers in Sokoto metropolis, Nigeria. Edorium J Mater Child Health.

